# (1*R*,2*R*,5*R*,6*S*,9*R*,10*S*,13*S*,14*S*,18*R*)-1,6,7,8,9,14,15,16,17,17,18-Undeca­chloro­penta­cyclo­[12.2.1.1^6,9^.0^2,13^.0^5,10^]octa­deca-7,15-diene

**DOI:** 10.1107/S160053680801622X

**Published:** 2008-06-13

**Authors:** Nicole Riddell, Robert McCrindle, Gilles Arsenault, Alan J Lough

**Affiliations:** a Wellington Laboratories, Research Division, Guelph, Ontario, Canada N1G 3M5; b Department of Chemistry, University of Guelph, Ontario, Canada N1G 2W1; c Department of Chemistry, University of Toronto, Ontario, Canada M5S 3H6

## Abstract

The title compound, C_18_H_13_Cl_11_, is an undecachlorinated commercial flame retardant. The asymmetric unit contains two independent half-mol­ecules. The complete mol­ecules are generated by crystallographic inversion symmetry, causing the terminal H atoms and one of the Cl atoms to be disordered equally over two sites in each mol­ecule. The central eight-membered rings are in chair-type conformations. In the crystal structure, there is a single weak inter­molecular C—H⋯Cl hydrogen bond.

## Related literature

For related literature, see: Riddell *et al.* (2008 ▶).
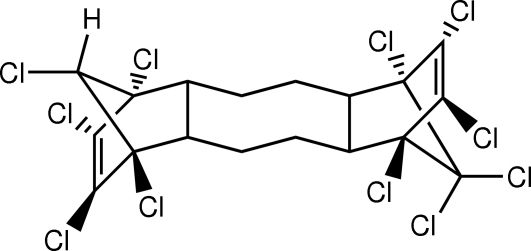


## Experimental

### 

#### Crystal data

C_18_H_13_Cl_11_
*M* *_r_* = 619.23
Monoclinic, 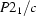

*a* = 13.3129 (5) Å
*b* = 12.1263 (6) Å
*c* = 14.7229 (7) Å
β = 99.505 (3)°
*V* = 2344.18 (18) Å^3^
*Z* = 4
Mo *K*α radiation
μ = 1.31 mm^−1^
*T* = 150 (1) K
0.26 × 0.20 × 0.15 mm

#### Data collection

Bruker–Nonius KappaCCD diffractometer
Absorption correction: multi-scan (*SORTAV*; Blessing, 1995 ▶) *T*_min_ = 0.715, *T*_max_ = 0.825
15654 measured reflections
5338 independent reflections
3481 reflections with *I* > 2σ(*I*)
*R*_int_ = 0.052

#### Refinement

*R*[*F* ^2^ > 2σ(*F* ^2^)] = 0.050
*wR*(*F* ^2^) = 0.118
*S* = 1.05
5338 reflections
272 parameters
H-atom parameters constrained
Δρ_max_ = 0.53 e Å^−3^
Δρ_min_ = −0.68 e Å^−3^

### 

Data collection: *COLLECT* (Nonius, 2002 ▶); cell refinement: *DENZO*–*SMN* (Otwinowski & Minor, 1997 ▶); data reduction: *DENZO*–*SMN*; program(s) used to solve structure: *SIR92* (Altomare *et al.*, 1994 ▶); program(s) used to refine structure: *SHELXTL* (Sheldrick, 2008 ▶); molecular graphics: *PLATON* (Spek, 2003 ▶); software used to prepare material for publication: *SHELXTL*.

## Supplementary Material

Crystal structure: contains datablocks global, I. DOI: 10.1107/S160053680801622X/pk2101sup1.cif

Structure factors: contains datablocks I. DOI: 10.1107/S160053680801622X/pk2101Isup2.hkl

Additional supplementary materials:  crystallographic information; 3D view; checkCIF report

## Figures and Tables

**Table 1:** Hydrogen-bond geometry (Å, °)

*D*—H⋯*A*	*D*—H	H⋯*A*	*D*⋯*A*	*D*—H⋯*A*
C1*B*—H1*B*⋯Cl4*A*^i^	1.00	2.70	3.656 (4)	160

Symmetry code: (i) 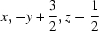.

**Fig. 1.**
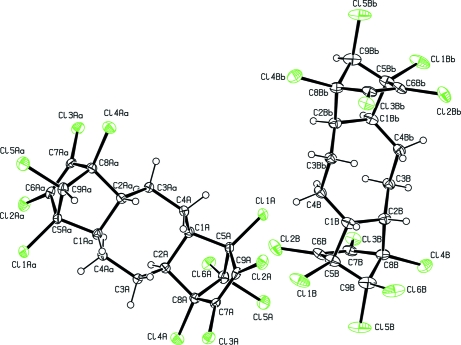
The asymmetric unit of the title compound. Displacement ellipsoids are drawn at the 30% probability level. The disorder is not shown and the atoms labeled with lower case suffixes a and b are related by the symmetry operators (-x, -y+1, -z+1) and (-x+1, - y+1, -z) respectively.

## supplementary crystallographic information

### Comment

For background information and related references see the previous paper (Riddell *et al.*, 2008). Dechlorane Plus (DP) is a commercial chlorinated flame retardant used in styrenic plastics (http://www.inchem.org/documents/ehc/ehc/ehc192.htm) to protect human life and property against fires. We have synthesized the dechlorinated compound (1R,2R,5R,6S,9R,10S,13S,14S,18R)-1,6,7,8,9,14,15,16,17,17,18- undecachloropentacyclo[12.2.1.1^6,9^.0^2,13^.0^5,10^]-octadeca-7,15-diene. GC/MS and ^1^H NMR spectroscopy have confirmed the basic structure of as having the DP-like structure with only 11 chlorine atoms. An NOE NMR experiment also strongly indicated that the proton on the bridging carbon atom was facing towards the cyclooctadiene ring since a positive through space interaction was observed. However, an X-ray structure determination was required to positively confirm the stereochemistry.

The asymmetric unit contains two independent half molecules. The symmetry complete molecules are generated by crystallographic inversion symmetry, causing atoms Cl6A and Cl6B, as well as the H atoms bonded to C9A and C9B to be disordered over two sites with equal occupancies. In both independent molecules the geometric parameters are the same within experimental error. The asymmetric unit is shown in Fig. 2. In the crystal structure there is a single weak intermolecular C—H···Cl interaction (Table 1).

### Experimental

The synthesis of the title compound was carried out at Wellington Laboratories using proprietary methods. The compound was isolated and purified using chromatographic techniques. For single-crystal *x*-ray crystallography, colourless crystals were grown from a solution in toluene.

### Refinement

All hydrogen atoms were placed in calculated positions with C—H distances of 0.99 and 1.00 Å and they were included in the refinement in a riding-model approximation with *U*_iso_ = 1.2*U*_eq_(C).

### Crystal data

**Table:**

C_18_H_13_Cl_11_	*F*_000_ = 1232
*M**_r_* = 619.23	*D*_x_ = 1.755 Mg m^−^^3^
Monoclinic, *P*2_1_/*c*	Mo *K*α radiation λ = 0.71073 Å
Hall symbol: -P 2ybc	Cell parameters from 15654 reflections
*a* = 13.3129 (5) Å	θ = 2.8–27.5º
*b* = 12.1263 (6) Å	µ = 1.31 mm^−^^1^
*c* = 14.7229 (7) Å	*T* = 150 (1) K
β = 99.505 (3)º	Block, colourless
*V* = 2344.18 (18) Å^3^	0.26 × 0.20 × 0.15 mm
*Z* = 4	

### Data collection

**Table:**

Bruker–Nonius KappaCCD diffractometer	5338 independent reflections
Radiation source: fine-focus sealed tube	3481 reflections with *I* > 2σ(*I*)
Monochromator: graphite	*R*_int_ = 0.052
Detector resolution: 9 pixels mm^-1^	θ_max_ = 27.5º
*T* = 150(2) K	θ_min_ = 2.8º
φ scans and ω scans with κ offsets	*h* = −17→17
Absorption correction: multi-scan(SORTAV; Blessing, 1995)	*k* = −14→15
*T*_min_ = 0.715, *T*_max_ = 0.825	*l* = −16→19
15654 measured reflections	

### Refinement

**Table:**

Refinement on *F*^2^	Hydrogen site location: inferred from neighbouring sites
Least-squares matrix: full	H-atom parameters constrained
*R*[*F*^2^ > 2σ(*F*^2^)] = 0.050	*w* = 1/[σ^2^(*F*_o_^2^) + (0.0314*P*)^2^ + 4.5718*P*] where *P* = (*F*_o_^2^ + 2*F*_c_^2^)/3
*wR*(*F*^2^) = 0.118	(Δ/σ)_max_ < 0.001
*S* = 1.05	Δρ_max_ = 0.53 e Å^−^^3^
5338 reflections	Δρ_min_ = −0.68 e Å^−^^3^
272 parameters	Extinction correction: SHELXTL (Sheldrick, 2008), Fc^*^=kFc[1+0.001xFc^2^λ^3^/sin(2θ)]^-1/4^
Primary atom site location: structure-invariant direct methods	Extinction coefficient: 0.0011 (3)
Secondary atom site location: difference Fourier map	

### Special details

**Table:**

Geometry. All e.s.d.'s (except the e.s.d. in the dihedral angle between two l.s. planes) are estimated using the full covariance matrix. The cell e.s.d.'s are taken into account individually in the estimation of e.s.d.'s in distances, angles and torsion angles; correlations between e.s.d.'s in cell parameters are only used when they are defined by crystal symmetry. An approximate (isotropic) treatment of cell e.s.d.'s is used for estimating e.s.d.'s involving l.s. planes.
Refinement. Refinement of *F*^2^ against ALL reflections. The weighted *R*-factor *wR* and goodness of fit *S* are based on *F*^2^, conventional *R*-factors *R* are based on *F*, with *F* set to zero for negative *F*^2^. The threshold expression of *F*^2^ > σ(*F*^2^) is used only for calculating *R*-factors(gt) *etc*. and is not relevant to the choice of reflections for refinement. *R*-factors based on *F*^2^ are statistically about twice as large as those based on *F*, and *R*- factors based on ALL data will be even larger.

### Fractional atomic coordinates and isotropic or equivalent isotropic displacement parameters (Å^2^)

**Table:**

	*x*	*y*	*z*	*U*_iso_*/*U*_eq_	Occ. (<1)
Cl1A	0.12630 (8)	0.59357 (8)	0.23239 (6)	0.0351 (3)	
Cl2A	−0.04105 (8)	0.77914 (9)	0.28215 (7)	0.0390 (3)	
Cl3A	0.03958 (8)	0.87394 (8)	0.49922 (7)	0.0371 (3)	
Cl4A	0.25958 (7)	0.75232 (9)	0.58340 (7)	0.0375 (3)	
Cl5A	0.28655 (8)	0.77926 (10)	0.35627 (7)	0.0449 (3)	
Cl6A	0.32477 (14)	0.57426 (16)	0.42481 (15)	0.0351 (5)	0.50
C1A	0.0896 (3)	0.5390 (3)	0.4083 (2)	0.0235 (8)	
H1A	0.1293	0.4710	0.3993	0.028*	
C2A	0.1294 (3)	0.5849 (3)	0.5085 (2)	0.0229 (8)	
H2A	0.1854	0.5355	0.5384	0.027*	
C3A	0.0525 (3)	0.5978 (3)	0.5746 (2)	0.0246 (8)	
H3A1	−0.0099	0.6331	0.5412	0.030*	
H3A2	0.0817	0.6477	0.6254	0.030*	
C4A	−0.0227 (3)	0.5119 (3)	0.3837 (2)	0.0268 (8)	
H4A1	−0.0412	0.5080	0.3158	0.032*	
H4A2	−0.0626	0.5724	0.4054	0.032*	
C5A	0.1236 (3)	0.6328 (3)	0.3464 (2)	0.0255 (8)	
C6A	0.0612 (3)	0.7346 (3)	0.3593 (3)	0.0263 (8)	
C7A	0.0923 (3)	0.7720 (3)	0.4438 (3)	0.0243 (8)	
C8A	0.1776 (3)	0.6973 (3)	0.4878 (2)	0.0245 (8)	
C9A	0.2268 (3)	0.6680 (3)	0.4039 (3)	0.0289 (9)	
H9C	0.2739	0.6037	0.4170	0.035*	0.50
Cl1B	0.50434 (8)	0.70192 (12)	0.26767 (8)	0.0573 (4)	
Cl2B	0.68792 (8)	0.51251 (11)	0.29107 (7)	0.0488 (3)	
Cl3B	0.83732 (7)	0.55922 (9)	0.12642 (7)	0.0400 (3)	
Cl4B	0.74566 (9)	0.77648 (10)	0.00260 (9)	0.0501 (3)	
Cl5B	0.70173 (9)	0.85653 (11)	0.21603 (10)	0.0647 (4)	
Cl6B	0.52623 (17)	0.8699 (2)	0.0840 (2)	0.0609 (8)	0.50
C1B	0.5100 (3)	0.6208 (3)	0.0915 (3)	0.0312 (9)	
H1B	0.4460	0.6647	0.0747	0.037*	
C2B	0.5781 (3)	0.6426 (3)	0.0152 (3)	0.0306 (9)	
H2B	0.5410	0.6952	−0.0308	0.037*	
C3B	0.6089 (3)	0.5433 (3)	−0.0368 (3)	0.0310 (9)	
H3B1	0.6333	0.4843	0.0079	0.037*	
H3B2	0.6663	0.5644	−0.0683	0.037*	
C4B	0.4792 (3)	0.5024 (3)	0.1091 (3)	0.0304 (9)	
H4B1	0.4586	0.4988	0.1706	0.037*	
H4B2	0.5394	0.4542	0.1104	0.037*	
C5B	0.5738 (3)	0.6757 (4)	0.1780 (3)	0.0362 (10)	
C6B	0.6701 (3)	0.6059 (4)	0.2039 (3)	0.0344 (10)	
C7B	0.7274 (3)	0.6241 (3)	0.1398 (3)	0.0324 (9)	
C8B	0.6712 (3)	0.7049 (3)	0.0715 (3)	0.0343 (9)	
C9B	0.6180 (3)	0.7760 (4)	0.1363 (3)	0.0444 (11)	
H9D	0.5630	0.8225	0.1008	0.053*	0.50

### Atomic displacement parameters (Å^2^)

**Table:**

	*U*^11^	*U*^22^	*U*^33^	*U*^12^	*U*^13^	*U*^23^
Cl1A	0.0514 (6)	0.0324 (5)	0.0245 (5)	−0.0025 (5)	0.0149 (4)	−0.0003 (4)
Cl2A	0.0392 (6)	0.0357 (6)	0.0398 (6)	0.0080 (5)	−0.0005 (4)	0.0127 (5)
Cl3A	0.0473 (6)	0.0243 (5)	0.0446 (6)	0.0049 (5)	0.0217 (5)	−0.0033 (4)
Cl4A	0.0297 (5)	0.0500 (7)	0.0331 (5)	−0.0137 (5)	0.0057 (4)	−0.0082 (5)
Cl5A	0.0406 (6)	0.0562 (7)	0.0428 (6)	−0.0200 (5)	0.0212 (5)	−0.0039 (5)
Cl6A	0.0289 (10)	0.0275 (10)	0.0519 (13)	0.0056 (8)	0.0154 (9)	0.0000 (9)
C1A	0.0272 (18)	0.0192 (19)	0.025 (2)	0.0036 (16)	0.0068 (15)	0.0029 (15)
C2A	0.0234 (18)	0.0228 (19)	0.0222 (19)	0.0048 (16)	0.0031 (15)	−0.0005 (15)
C3A	0.0278 (19)	0.024 (2)	0.0225 (19)	−0.0043 (16)	0.0055 (15)	0.0007 (16)
C4A	0.034 (2)	0.026 (2)	0.0194 (19)	−0.0004 (17)	0.0010 (16)	0.0025 (16)
C5A	0.033 (2)	0.024 (2)	0.0217 (19)	0.0011 (17)	0.0105 (16)	0.0037 (16)
C6A	0.0250 (19)	0.024 (2)	0.031 (2)	−0.0031 (16)	0.0074 (16)	0.0079 (16)
C7A	0.0302 (19)	0.0164 (18)	0.029 (2)	−0.0018 (16)	0.0139 (16)	−0.0012 (15)
C8A	0.0234 (18)	0.028 (2)	0.0231 (19)	−0.0042 (16)	0.0059 (15)	−0.0017 (16)
C9A	0.0264 (19)	0.031 (2)	0.031 (2)	0.0052 (17)	0.0111 (16)	0.0046 (17)
Cl1B	0.0337 (6)	0.0861 (10)	0.0536 (8)	0.0007 (6)	0.0119 (5)	−0.0381 (7)
Cl2B	0.0441 (6)	0.0722 (9)	0.0271 (6)	0.0022 (6)	−0.0024 (5)	0.0005 (5)
Cl3B	0.0233 (5)	0.0504 (7)	0.0458 (6)	0.0043 (5)	0.0042 (4)	−0.0118 (5)
Cl4B	0.0484 (7)	0.0451 (7)	0.0570 (8)	−0.0176 (6)	0.0088 (5)	−0.0010 (6)
Cl5B	0.0413 (6)	0.0622 (8)	0.0884 (10)	−0.0096 (6)	0.0042 (6)	−0.0458 (8)
Cl6B	0.0358 (12)	0.0373 (13)	0.100 (2)	0.0108 (10)	−0.0158 (12)	−0.0205 (13)
C1B	0.0195 (18)	0.040 (2)	0.032 (2)	0.0056 (18)	−0.0011 (16)	−0.0101 (19)
C2B	0.0257 (19)	0.030 (2)	0.034 (2)	0.0015 (17)	−0.0028 (17)	−0.0019 (17)
C3B	0.0255 (19)	0.038 (2)	0.028 (2)	−0.0051 (18)	0.0023 (16)	−0.0041 (18)
C4B	0.0238 (19)	0.042 (2)	0.025 (2)	−0.0002 (18)	0.0016 (15)	−0.0035 (18)
C5B	0.0232 (19)	0.048 (3)	0.038 (2)	0.0026 (19)	0.0056 (17)	−0.016 (2)
C6B	0.027 (2)	0.050 (3)	0.024 (2)	0.0022 (19)	−0.0012 (16)	−0.0133 (19)
C7B	0.0218 (19)	0.041 (2)	0.033 (2)	0.0013 (18)	−0.0002 (16)	−0.0141 (19)
C8B	0.029 (2)	0.033 (2)	0.039 (2)	−0.0032 (18)	0.0017 (18)	−0.0076 (19)
C9B	0.031 (2)	0.041 (3)	0.058 (3)	0.006 (2)	−0.004 (2)	−0.017 (2)

### Geometric parameters (Å, °)

**Table:**

Cl1A—C5A	1.751 (4)	Cl1B—C5B	1.761 (4)
Cl2A—C6A	1.710 (4)	Cl2B—C6B	1.698 (4)
Cl3A—C7A	1.696 (4)	Cl3B—C7B	1.701 (4)
Cl4A—C8A	1.763 (4)	Cl4B—C8B	1.760 (4)
Cl5A—C9A	1.769 (4)	Cl5B—C9B	1.772 (4)
Cl6A—C9A	1.720 (4)	Cl6B—C9B	1.752 (5)
C1A—C4A	1.515 (5)	C1B—C4B	1.527 (6)
C1A—C5A	1.570 (5)	C1B—C5B	1.559 (5)
C1A—C2A	1.584 (5)	C1B—C2B	1.579 (5)
C1A—H1A	1.0000	C1B—H1B	1.0000
C2A—C3A	1.533 (5)	C2B—C3B	1.518 (5)
C2A—C8A	1.558 (5)	C2B—C8B	1.567 (5)
C2A—H2A	1.0000	C2B—H2B	1.0000
C3A—C4A^i^	1.544 (5)	C3B—C4B^ii^	1.551 (5)
C3A—H3A1	0.9900	C3B—H3B1	0.9900
C3A—H3A2	0.9900	C3B—H3B2	0.9900
C4A—C3A^i^	1.544 (5)	C4B—C3B^ii^	1.551 (5)
C4A—H4A1	0.9900	C4B—H4B1	0.9900
C4A—H4A2	0.9900	C4B—H4B2	0.9900
C5A—C6A	1.518 (5)	C5B—C9B	1.524 (7)
C5A—C9A	1.548 (5)	C5B—C6B	1.531 (6)
C6A—C7A	1.324 (5)	C6B—C7B	1.328 (5)
C7A—C8A	1.512 (5)	C7B—C8B	1.511 (6)
C8A—C9A	1.533 (5)	C8B—C9B	1.542 (6)
			
C4A—C1A—C5A	112.7 (3)	C4B—C1B—C5B	112.8 (3)
C4A—C1A—C2A	117.6 (3)	C4B—C1B—C2B	118.7 (3)
C5A—C1A—C2A	101.6 (3)	C5B—C1B—C2B	102.2 (3)
C4A—C1A—H1A	108.1	C4B—C1B—H1B	107.5
C5A—C1A—H1A	108.1	C5B—C1B—H1B	107.5
C2A—C1A—H1A	108.1	C2B—C1B—H1B	107.5
C3A—C2A—C8A	112.0 (3)	C3B—C2B—C8B	113.2 (3)
C3A—C2A—C1A	118.2 (3)	C3B—C2B—C1B	117.4 (3)
C8A—C2A—C1A	102.2 (3)	C8B—C2B—C1B	101.6 (3)
C3A—C2A—H2A	108.0	C3B—C2B—H2B	108.1
C8A—C2A—H2A	108.0	C8B—C2B—H2B	108.1
C1A—C2A—H2A	108.0	C1B—C2B—H2B	108.1
C2A—C3A—C4A^i^	114.1 (3)	C2B—C3B—C4B^ii^	113.1 (3)
C2A—C3A—H3A1	108.7	C2B—C3B—H3B1	109.0
C4A^i^—C3A—H3A1	108.7	C4B^ii^—C3B—H3B1	109.0
C2A—C3A—H3A2	108.7	C2B—C3B—H3B2	109.0
C4A^i^—C3A—H3A2	108.7	C4B^ii^—C3B—H3B2	109.0
H3A1—C3A—H3A2	107.6	H3B1—C3B—H3B2	107.8
C1A—C4A—C3A^i^	113.6 (3)	C1B—C4B—C3B^ii^	114.4 (3)
C1A—C4A—H4A1	108.8	C1B—C4B—H4B1	108.7
C3A^i^—C4A—H4A1	108.8	C3B^ii^—C4B—H4B1	108.7
C1A—C4A—H4A2	108.8	C1B—C4B—H4B2	108.7
C3A^i^—C4A—H4A2	108.8	C3B^ii^—C4B—H4B2	108.7
H4A1—C4A—H4A2	107.7	H4B1—C4B—H4B2	107.6
C6A—C5A—C9A	99.3 (3)	C9B—C5B—C6B	100.3 (3)
C6A—C5A—C1A	107.5 (3)	C9B—C5B—C1B	101.9 (3)
C9A—C5A—C1A	101.4 (3)	C6B—C5B—C1B	106.8 (3)
C6A—C5A—Cl1A	116.0 (3)	C9B—C5B—Cl1B	116.3 (3)
C9A—C5A—Cl1A	116.2 (2)	C6B—C5B—Cl1B	115.8 (3)
C1A—C5A—Cl1A	114.4 (3)	C1B—C5B—Cl1B	114.0 (3)
C7A—C6A—C5A	107.6 (3)	C7B—C6B—C5B	106.7 (4)
C7A—C6A—Cl2A	127.4 (3)	C7B—C6B—Cl2B	128.5 (3)
C5A—C6A—Cl2A	124.5 (3)	C5B—C6B—Cl2B	124.3 (3)
C6A—C7A—C8A	107.1 (3)	C6B—C7B—C8B	107.5 (3)
C6A—C7A—Cl3A	127.7 (3)	C6B—C7B—Cl3B	127.8 (4)
C8A—C7A—Cl3A	124.8 (3)	C8B—C7B—Cl3B	124.4 (3)
C7A—C8A—C9A	100.6 (3)	C7B—C8B—C9B	100.3 (3)
C7A—C8A—C2A	107.6 (3)	C7B—C8B—C2B	107.5 (3)
C9A—C8A—C2A	101.5 (3)	C9B—C8B—C2B	101.2 (3)
C7A—C8A—Cl4A	116.0 (3)	C7B—C8B—Cl4B	116.0 (3)
C9A—C8A—Cl4A	116.2 (3)	C9B—C8B—Cl4B	116.2 (3)
C2A—C8A—Cl4A	113.3 (3)	C2B—C8B—Cl4B	113.8 (3)
C8A—C9A—C5A	92.6 (3)	C5B—C9B—C8B	92.9 (3)
C8A—C9A—Cl6A	114.9 (3)	C5B—C9B—Cl6B	114.1 (3)
C5A—C9A—Cl6A	119.6 (3)	C8B—C9B—Cl6B	116.7 (3)
C8A—C9A—Cl5A	115.0 (3)	C5B—C9B—Cl5B	114.6 (3)
C5A—C9A—Cl5A	114.2 (3)	C8B—C9B—Cl5B	114.5 (3)
Cl6A—C9A—Cl5A	101.4 (2)	Cl6B—C9B—Cl5B	104.5 (2)

Symmetry codes: (i) −*x*, −*y*+1, −*z*+1; (ii) −*x*+1, −*y*+1, −*z*.

### Hydrogen-bond geometry (Å, °)

**Table:**

*D*—H···*A*	*D*—H	H···*A*	*D*···*A*	*D*—H···*A*
C1B—H1B···Cl4A^iii^	1.00	2.70	3.656 (4)	160

Symmetry codes: (iii) *x*, −*y*+3/2, *z*−1/2.
